# Comparison of Peptidomes Extracted from Healthy Tissue and Tumor Tissue of the Parotid Glands and Saliva Samples

**DOI:** 10.3390/ijms25168799

**Published:** 2024-08-13

**Authors:** Michał Puchalski, Dmitry Tretiakow, Andrzej Skorek, Konrad Szydłowski, Dominik Stodulski, Bogusław Mikaszewski, Amadeusz Odroniec, Natalia Musiał, Marcel Thiel, Paulina Czaplewska, Stanisław Ołdziej

**Affiliations:** 1 Intercollegiate Faculty of Biotechnology UG&MUG, University of Gdańsk, Abrahama 58, 80-307 Gdańsk, Poland; michal.puchalski@phdstud.ug.edu.pl (M.P.); amadi0909@gmail.com (A.O.); natalia.musial@phdstud.ug.edu.pl (N.M.); marcel.thiel@ug.edu.pl (M.T.); paulina.czaplewska@ug.edu.pl (P.C.); 2 Department of Otolaryngology, the Nicolaus Copernicus Hospital in Gdansk, Copernicus Healthcare Entity, Powstańców Warszawskich 1/2, 80-152 Gdansk, Poland; andrzej.skorek@gumed.edu.pl (A.S.); konrad.szyd@gmail.com (K.S.); 3 Department of Otolaryngology, Faculty of Medicine, Medical University of Gdansk, Smoluchowskiego 17, 80-214 Gdansk, Poland; dstodulski@gumed.edu.pl (D.S.); boguslaw.mikaszewski@gumed.edu.pl (B.M.)

**Keywords:** salivary gland neoplasms, saliva, biomarkers, peptidome, mass spectrometry

## Abstract

Salivary gland tumors are highly variable in clinical presentation and histology. The World Health Organization (WHO) classifies 22 types of malignant and 11 types of benign tumors of the salivary glands. Diagnosis of salivary gland tumors is based on imaging (ultrasound, magnetic resonance imaging) and fine-needle aspiration biopsy, but the final diagnosis is based on histopathological examination of the removed tumor tissue. In this pilot study, we are testing a new approach to identifying peptide biomarkers in saliva that can be used to diagnose salivary gland tumors. The research material for the peptidomic studies was extracts from washings of neoplastic tissues and healthy tissues (control samples). At the same time, saliva samples from patients and healthy individuals were analyzed. The comparison of the peptidome composition of tissue extracts and saliva samples may allow the identification of potential peptide markers of salivary gland tumors in patients’ saliva. The peptidome compositions extracted from 18 tumor and 18 healthy tissue samples, patients’ saliva samples (11 samples), and healthy saliva samples (8 samples) were analyzed by LC-MS tandem mass spectrometry. A group of 109 peptides was identified that were present only in the tumor tissue extracts and in the patients’ saliva samples. Some of the identified peptides were derived from proteins previously suggested as potential biomarkers of salivary gland tumors (ANXA1, BPIFA2, FGB, GAPDH, HSPB1, IGHG1, VIM) or tumors of other tissues or organs (SERPINA1, APOA2, CSTB, GSTP1, S100A8, S100A9, TPI1). Unfortunately, none of the identified peptides were present in all samples analyzed. This may be due to the high heterogeneity of this type of cancer. The surprising result was that extracts from tumor tissue did not contain peptides derived from salivary gland-specific proteins (STATH, SMR3B, HTN1, HTN3). These results could suggest that the developing tumor suppresses the production of proteins that are essential components of saliva.

## 1. Introduction

Neoplasms of the major salivary glands account for approximately 3% of all tumors in the head and neck region. Most tumors develop in the parotid gland (80%), of which 4/5 are benign lesions [1,2]. These tumors are very different regarding their histology and clinical course. The World Health Organization (WHO) classification distinguishes 22 types of malignant and 11 benign tumors originating from the salivary glands [3]. Diagnostics of salivary gland tumors are based on imaging (ultrasound, magnetic resonance imaging) and fine-needle aspiration biopsy (FNA); however, the final diagnosis is based on the histopathological examination of the removed tumor [2,4]. The most common treatment for salivary gland tumors is surgical removal and complementary radiotherapy in the case of cancer detection. Removal of the parotid gland is always connected with a risk of damaging the facial nerve, which causes significant functional disorders (disorders of the mouth, eyes closing) and cosmetic defects. In the case of benign tumors, the priority is to maintain the anatomical continuity of the facial nerve. In the case of a malignant tumor, its radical removal is the most important, even with the sacrifice of the facial nerve [2,5,6]. Therefore, preoperative diagnostics differentiating malignant from benign changes are crucial. The prognosis for benign neoplasms is excellent, as the recurrence rate is about 1–2%. Disease-free 5-year survival is observed in about 60% of malignant parotid tumor cases and depends on their stage and grade [2,7]. The signs and symptoms indicating malignancy of salivary gland tumors (e.g., facial nerve palsy, skin infiltration) are related to the highest stage of disease and poor prognosis. Clinical picture, modern imaging techniques, and FNA do not allow distinguishing between benign and malignant tumors in every case; thus, other diagnostic methods (i.e., based on saliva testing) are developed [8].

Due to their rarity and high heterogeneity, salivary gland tumors remain relatively poorly characterized in terms of molecular analysis; most of the studies on this topic have focused on the genetic aspect of tumorigenesis [9], demonstrating, among other things, the presence of characteristic fusion oncogenes in salivary gland tumors [10]. Recently, there has been a gradual increase in interest in proteomic studies using mass spectrometric methods as a source of new tumor biomarkers [11]. Although various proteins [12,13,14,15] have been proposed as potential biomarkers for different salivary gland tumors, their diagnostic efficacy has not been confirmed [11]. No study has been published to date analyzing salivary gland tumors at the peptidomic level. The peptidome—consisting of thousands of peptides: remnants of protein degradation; specific precursor-derived peptides such as hormones, cytokines, or growth factors; and translation products of small open reading frames (smORFs) [16]—responds dynamically to changes in the body, providing a rich source of potential biomarkers. This results in the rapid expansion of peptidomics research observed in recent years [17,18], demonstrating characteristic peptidomic patterns of many diseases, including various types of cancer [19,20,21]. Furthermore, it has been shown that peptidomic analyses can detect statistically significant differences observed in bottom-up proteomics [22].

Proteomic studies of salivary tumor tissues known from the literature used either homogenized frozen [15] or FFPE tissues [12,23]. Homogenized tissues are a commonly used material in proteomic research. However, their major disadvantage is the presence of many proteins (some of these are in high concentrations) from the examined tissue, making it challenging to identify potential biomarkers. An interesting solution that allows the analysis of tissues derived from tumor tissues of the salivary glands without the need to use homogenates is the method of analyzing the composition of the FNA fluid (a saline wash of a needle after the fine needle aspiration of a tumor) [12,13]. Using the FNA fluid as a source of proteins/peptides deserves attention, given the widespread use of FNA in diagnosing salivary gland tumors. The problems related to the FNA fluid analysis are the minimal amount of the sample and the problem related to the FNA procedure itself, i.e., the high probability of collecting the sample from the tissue not covered by the tumor.

The purpose of the present study was to identify specific peptides secreted into the saliva of patients with salivary gland cancer. The possible identification of peptide markers of tumor tissue could be used to develop a non-invasive method of salivary gland tumor detection.

## 2. Results

### 2.1. Peptide Extraction Methods and Sample Handling

In our experiments we used fresh or frozen (samples are referred to as fresh or frozen) salivary gland tissue samples, healthy or tumor. Peptides were extracted from the tissue samples using phosphate buffer (PBS extracts) or 0.1% trifluoroacetic acid solution in water (TFA extracts). A more detailed description of sample preparation is provided in the Section 4.

We analyzed tissue samples from 14 patients. In addition, we analyzed saliva samples from 8 healthy donors (control) and 11 patients. Information on the samples analyzed as well as the handling of the tissue samples (frozen or fresh) and the peptide extraction methods are summarized in Table 1. Patient information is presented in Table S1. Detailed information on each of the extracts used in the study is provided in Table S2.

The first step of our research was to compare the peptidome composition extracted from tissue samples obtained by two different extraction methods: (i) extraction with PBS alone (referred to in the text as the PBS method); (ii) extraction with 0.1% TFA solution following PBS extraction (referred to in the text as the TFA method). Twenty PBS extracts and 16 TFA extracts from different samples were used in the analysis. A total of 7771 peptides were identified in all extracts obtained with PBS extracts, more than twice as many as in the TFA extracts, where only 3540 peptides were identified. The two types of extracts also differed significantly in composition, with only about 9% (931 out of 10,380) of all unique peptides identified in both PBS and TFA extracts. The PBS extracts (bearing in mind that the PBS wash was performed first) were richer in peptides, but the extraction with TFA allowed the isolation of a different fraction of peptides. On average, about 388 and 221 unique peptides were identified in the PBS and TFA extract samples, respectively. On the other hand, we tested the effect of sample handling on peptide extraction by comparing extracts obtained from frozen and fresh salivary gland samples. A total of 10,380 unique peptides were identified in all samples (Table S3), with an average of approximately 288 unique peptides per sample. Similar numbers of peptides were identified in both extracts (6751 in fresh and 6913 in frozen). Almost one-third of all identified peptides (3284 out of 10,380) were common to both fresh and frozen tissue extracts. The remaining peptides were clustered into two almost equal sets (3467 and 3629 peptides), also close to 1/3 of the total pool, containing peptides unique to either fresh or frozen tissue extracts. Thus, although fresh and frozen salivary gland tissue extracts are similar in terms of the number of peptides identified, they differ significantly in terms of peptidome composition. The results of the experiments shown above indicate that there is no best sample preparation or extraction method in terms of the number of unique peptides identified. Each of the analysis options shown above yields a significant number of unique peptides specific to the type of sample preparation.

### 2.2. Comparison of the Peptidome Composition of Salivary Gland Extracts and Saliva

**Figure 1:**
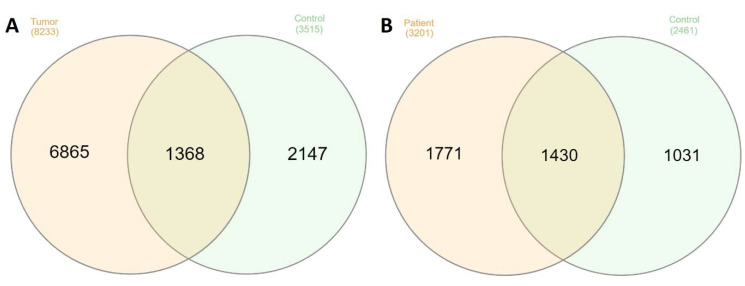
Venn diagrams showing numbers of identified peptides: (**A**) peptides identified in extracts from tumor tissue and healthy salivary gland samples, (**B**) peptides identified in extracts from patients’ and healthy subjects’ saliva. Diagrams show combined results from analyzing all samples.

To investigate differences in the peptidome of salivary gland tumors and healthy tissue, we compared extracts (both PBS and TFA) obtained from tumor tissue and extracts from healthy control tissue (Figure 1A). The extracts from tumor tissue were significantly richer in peptides, with 8233 unique peptides identified, compared to only 3515 peptides identified in the control samples. Surprisingly only a small number of peptides (1368) were shared between the tumor and healthy tissue (see Figure 1A). We analyzed saliva samples in a similar manner. The study used saliva samples from 8 healthy subjects (control) and 11 patients. In total, 3272 peptides were identified in patients’ saliva, compared to 2461 peptides identified in control samples. We found that 1474 peptides were identical for both sample types, while 1798 peptides were found only in patients’ saliva (see Figure 1B). The data presented in Figure 1 clearly show that both tumor tissue extracts and patients’ saliva contain a much higher number of unique peptides. The increased number of peptides present in tumor-associated samples may be related to the increased proteolytic activity in tumor tissue postulated in the literature [24]. It should be noted, however, that the peptides observed in saliva result from the proteolytic activity of two distinct biological systems, namely human proteases as well as the proteases of oral microbiota which make in-depth analysis (identification of possible enzymes involved in protein degradation) impossible. Two methods were used to isolate the peptide fraction: “Direct method” and SPE extraction (see Materials and Methods section). SPE extraction resulted in a significantly higher number of identified peptides, so all patients’ samples underwent this procedure.

### 2.3. Bioinformatic Analysis

**Figure 2:**
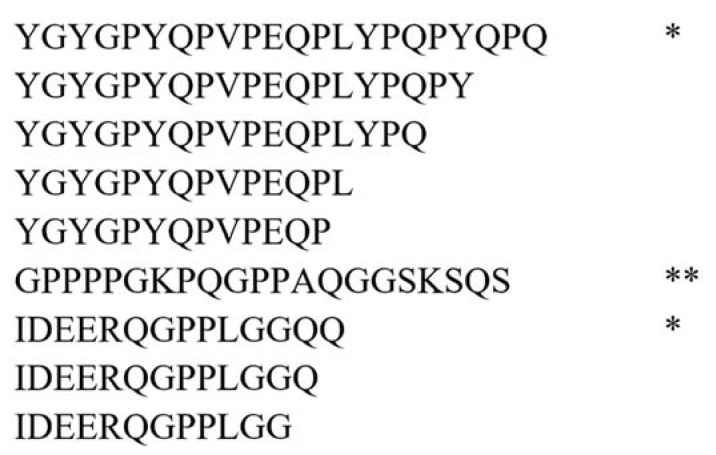
An example of the classification of identified peptides; *—unique peptide, **—stand-alone peptide.

In the search for potential biomarkers of salivary gland cancer, peptides with overlapping sequences are of little interest. We compared the peptides identified in each of the analyses (see Section 2.2) to find overlapping sequences and selected only those not contained in the sequences of other peptides as unique peptides. In addition, we also identified stand-alone peptides that were not included in the sequences of others and did not contain any identified peptides themselves (see Figure 2). As shown in Figure 2, peptides not marked * or ** were not considered in further analysis.

Table 2 provides numbers of all peptides identified in each analysis, categorized into unique and stand-alone statuses. Less than half of all identified peptides could be classified as unique peptides and 10–20% as stand-alone peptides. There are no significant differences in the content of unique and stand-alone peptides between tumor samples and controls and between different extraction methods. However, we observed noticeable differences between the peptide composition of samples from salivary glands and saliva. Unique and stand-alone peptides accounted for 46.9% (4869 of 10,380) and 20% (2077 of 10,380) of all peptides identified in salivary gland samples, respectively. For saliva samples, the values are much lower, at 35% for unique peptides (1490 of 4259) and 10% for stand-alone peptides (420 of 4259). The lower number of unique and stand-alone peptides identified in saliva samples is probably a result of higher proteolytic activity in saliva caused by the presence of a large number of microorganisms and their proteases in the oral cavity.

The next step was to look at peptides identified in saliva and compare them with peptide sequences from salivary gland extracts. We assumed that intense proteolytic enzyme activity occurs in human saliva, so we focused on looking for saliva peptides whose sequences are identical to peptides identified in salivary gland extracts (common peptides) or entirely contained within the sequences of longer peptides of salivary gland origin (overlapping sequences). The results of this analysis are presented in Table 3.

We analyzed the peptide data sets obtained in the study (e.g., Saliva patient) and the sets created by excluding peptides in common with the control (e.g., Saliva patient only). The comparison of peptides specific to patients’ saliva (Saliva patient only) with peptides specific to salivary gland tumors (SG tumor only) is particularly noteworthy. The peptides identified in this analysis may be potential biomarkers to characterize salivary gland tumors. We found 16 peptides that are common to both data sets compared (Saliva patient only and SG tumor only), but also 109 peptides identified in saliva whose sequences were contained within the sequences of longer peptides identified in salivary gland tumor extracts. Sequences of peptides identified in the group Saliva patient only—SG tumor only (see Table 3) are shown in Table S4.

### 2.4. Possible Peptide Biomarkers of Salivary Gland Tumors

Our research focuses on peptidomic characterization of salivary gland tumors, but it is challenging to analyze peptides in isolation from the parent protein. Therefore, the next step was to look at the proteins from which the peptides were identified as observed only in patients’ saliva samples and extracts from tumor tissue (Saliva patient only—SG tumor only in Table 3). In our study, we identified 109 peptides uniquely found only in tumor tissue extracts and patients’ saliva. Table 4 lists the proteins from which the selected peptides are derived, and Table S4 (Supplemental Data) lists the amino acid sequences of these peptides.

### 2.5. Saliva-Specific Proteins

**Figure 3:**
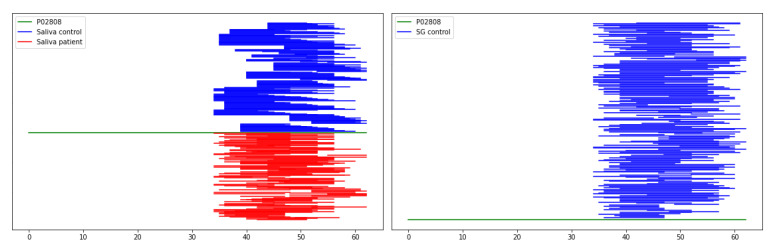
Coverage of the amino acid sequence of statherin (UniProt ID: P02808) by peptides identified in saliva (**left**) and tissue extracts (**right**). The green line schematically shows the amino acid sequence of statherin, the *x*-axis shows the length of the protein. In both panels, red and blue lines represent peptides identified as fragments of the parent protein. Blue colors represent peptides identified in saliva from healthy individuals and peptides extracted from healthy tissue samples. Peptides identified in patients’ saliva or extracted from tumor tissue are shown in red.

Analyzing the peptidome of tumor tissue extracts, we observed a complete absence of peptides derived from four saliva-specific proteins. As shown in Figure 3, extracts derived from tumor tissue do not contain peptides derived from statherin (STATH) and three other proteins: submaxillary gland androgen-regulated protein 3b (SMR3B), histatin 1 (HTN1), and histatin 3 (HTN3). Peptides derived from these four proteins are abundantly represented in healthy tissue extracts and saliva samples (both patient and control). To ensure the quality of identification we compared our results with reference data from the Peptide Atlas database [25]. In the case of histatin 1 and histatin 3, we could identify only one peptide belonging to those proteins in one extract sample from tumor tissue; however, we suspect that such identification was caused by the imperfect separation of healthy tissue from tumor.

## 3. Discussion

The most interesting results with possible diagnostic implications are the peptides identified only in the tissues of tumor tissue extracts and found in the patient’s saliva (see Table 4 and Table S4). Two buffers (PBS and TFA) were used to extract peptides from tissues, and analyses were performed on fresh and frozen samples. We observed that out of 109 selected peptides (Table 4), 104 were identified in the frozen samples, while only 88 peptides were identified in the fresh samples. Based on these preliminary results, a more promising strategy seems to be the analysis of the frozen samples. This observation also has a purely practical dimension. The use of frozen samples allows for an efficient organization of experiments, which is extremely difficult when analyzing fresh samples. As for the buffers’ effectiveness: 104 out of 109 selected peptides were identified in PBS extracts and only 79 in TFA extracts. PBS extraction is the first stage of the procedure, so necessarily the most peptides are found there. However, most of these peptides originate from blood and are not specific to the tissues being analyzed. It should be noted, however, that TFA extracts contain peptides unique to this fraction, so it seems reasonable to use multi-step extraction with perhaps other buffers in further studies. It should be noted that various types of acidic buffers have long been used to extract potentially immunogenic peptides that may be used in anti-cancer therapies [26,27].

All 109 peptides shown in Table 4 were assigned to 24 protein groups. Typical saliva-specific proteins (marked white in Table 4) were the most heavily represented, accounting for nearly half of all identified peptides. Those peptides are fragments of basic salivary proline-rich proteins (32 peptides) or salivary acidic proline-rich phosphoprotein (17 peptides), and, in addition, one peptide belonging to the parotid secretory SPLUNC2 (short palate, lung, and nasal epithelium clone 2). Among other proteins, relatively many peptides were identified for actin (nine peptides), histones (nine peptides), hemoglobin (five peptides), and keratin (four peptides). These proteins (marked white in Table 4), and consequently the peptides derived from them, are commonly found in many tissues, so, like saliva-specific proteins, they do not appear to be particularly interesting from a diagnostic point of view.

At least seven (marked in red in Table 4) of these 15 proteins have previously been shown to have differential expression in salivary gland tumors: annexin A1 (ANXA1), glyceraldehyde-3-phosphate dehydrogenase (GAPDH), fibrinogen beta chain (FBG), heat shock protein beta-1 (HSPB1), immunoglobulin gamma-1 chain C region (IGHG1), BPI fold-containing family A member 2 (BPIFA2), and vimentin (VIM). Donadio et al. [13] observed that ANXA1 and GAPDH are upregulated in pleomorphic adenomas and FBG, HSPB1, and IGHG1 are upregulated in Warthin’s tumors. Also, Seccia et al. [12] observed upregulation of ANXA1 in pleomorphic adenomas and IGHG1 in Warthin’s tumors, while Mutlu et al. [15] observed upregulation of VIM and downregulation of FGB in pleomorphic adenomas patients. Pereira et al. [28] suggested BPIFA2 as a potential biomarker for salivary mucoepidermoid carcinomas. González-Arriagada et al. [29] showed that BPIFA2 level in head and neck cancer decreases after radiotherapy. However, it should be mentioned that to date, studies on the proteome/peptidome of salivary gland tumors are few and far between and the scope of research conducted varies widely from studies aimed at diagnosis [12,13,14,15] to assessment of the effects of the radiation therapy [29]. Thus far, the results of these studies have not found a practical diagnostic application.

To our knowledge, the remaining proteins (marked yellow in Table 4) have not been associated with salivary gland tumors but have been linked to other types of cancer. Overexpression of alpha-1-antitrypsin (SERPINA1) promotes tumor progression in colorectal [30] and gastric [31] cancer, and its high level in plasma was observed in lung and prostate cancer patients [32]. Apolipoprotein A-II (APOA2) was proposed as a potential marker for urinary bladder [33] and pancreatic cancer [34]. Cystatin B was proposed as a biomarker in various tumors like bladder and ovarian cancer [35,36]. Glutathione S-transferase P (GSTP1) is downregulated in prostate cancer and upregulated in many cancer types, e.g., colorectal, thyroid, or breast cancer [37]. Also, a high level of glyceraldehyde-3-phosphate dehydrogenase (GAPDH) was found in various cancer types [38]. AHNAK was proposed as a biomarker for bladder urothelial carcinoma [39] and is strictly related to cell migration in mesothelioma [40]. Calprotectin, a heterodimer formed from the combination of S100A8 and S100A9 proteins, is commonly upregulated in many tumors and likely plays a critical (essential) role in inflammation-associated cancers [41]. Protein-glutamine gamma-glutamyltransferase E (TGM3) was proposed as a marker for some head and neck cancer types [42,43]. Thymosin beta-4 (TMSB4X) is overexpressed in pancreatic cancer [44] and was proposed as a biomarker for colorectal cancer [45]. Triosephosphate isomerase (TPI1) is upregulated in neoplasms like gastric or breast cancer and is related to cell migration and invasion [46,47].

Each of the peptides identified exclusively in tumor tissue extracts was only found in a maximum of 2–3 samples analyzed. This state of affairs may be related to the remarkable heterogeneity of salivary gland tumors [3]. Most of the samples examined in this work were from so-called mixed tumors (see Table S1). The results were obtained on a relatively small number of samples (14 patients) (see Table 1). The tumor tissue samples used for the study were derived from different types of tumors and different stages of malignancy (see Table S1). In addition, all data analyses were performed collectively for all available data and not for individual samples. The obtained results (see Table 4), despite the limitations presented above, are promising and allow for the planning of more targeted studies on a larger group of patients, more focused on taking into account the type and development stage of salivary gland tumors.

Using the OpenProt library [48] to identify proteins/peptides gives exciting and promising results. However, as mentioned in the Introduction, this is a pilot study to show that the proposed new methodological solutions (peptide extraction, use of the OpenProt library) are applicable to the planned research. Using the OpenProt library does not generate additional costs and allows us to increase the number of identified peptides by a few percent. As shown in Table 4, 3 out of 30 (10%) of the identified potential biomarkers were identified thanks to the OpenProt database [48]. These peptides would be omitted if classical UniProt [49] or NCBI [50] databases were used in searches. It should be noted however, that recent editions of the UniProt database [49] go in the direction of including not only the so-called canonical sequences, but also protein isoforms, which means that the content of the UniProt database also begins to include some of the information available only in the OpenProt database [48,51]. The reason for that could be that non-canonical products of eukaryotic genes (isoforms, pseudogenes) are of increasing interest in studies of tumorigenesis [52,53].

As outlined in Section 2.5. no peptides derived from four saliva-specific proteins (SATH, SMR3B, HTN1, HTN3) were identified in extracts from tumor tissue. It is very interesting that all four proteins mentioned above are specifically produced in salivary glands [54] and their genes are located on chromosome 4 in a very close proximity (www.ncbi.nlm.nih.gov/gene/3347, accessed on 15 June 2024; www.ncbi.nlm.nih.gov/gene/3346, accessed on 15 June 2024; www.ncbi.nlm.nih.gov/gene/6779, accessed on 15 June 2024; www.ncbi.nlm.nih.gov/gene/10879, accessed on 15 June 2024). The lack of identification of peptides from proteins STATH, SMR3B, HTN1, and HTN3 in extracts from tumor tissues may suggest that there is a complete suppression of the production of these proteins in tumor tissues. Peptides derived from other proteins produced in salivary glands, e.g., basic salivary proline-rich protein 2, are observed in all types of samples (healthy tissue, cancerous tissue), but the gene encoding this protein (PRB2) is located on chromosome 12. It is of great interest that salivary gland tumors are extremely diverse [3] and in our study samples from different types of tumors were used (see Table S1). However, regardless of the tumor type, the effect is the same: a lack of occurrence of peptides derived from proteins STATH, SMR3B, HTN1, and HTN3. The fact that peptides from these proteins are present in the saliva of patients with detected cancer is related to the fact that not all salivary glands are occupied by cancer, so the remaining healthy glands are able to produce the proteins in question. The result obtained is extremely interesting for several reasons. Firstly, it may have enormous diagnostic significance even with current methods based on fine-needle biopsies after using appropriate antibodies to pick out healthy tissue (presence of proteins) and no staining with tumor tissue (absence of proteins). With the heterogeneity of salivary gland tumors, a method capable of distinguishing healthy tissue from neoplastic tissue would be extremely valuable. Secondly, if the expression of a specific number of genes is stopped during the tumorigenesis process, then perhaps further research would lead to the description of the mechanism of this process, which in the long run would open a way to new diagnostic tests and/or therapeutic methods.

## 4. Materials and Methods

### 4.1. Salivary Gland Tissue Collection

A fragment of the tumor and salivary gland tissue was collected after parotidectomy. Some samples were stored at 4 °C after the surgery procedure, and peptide extraction was processed on the same day (those samples are called Fresh). The remaining samples were placed in a freezer at a temperature of −20 °C within 4 h of collection and processed later (those samples are called Frozen). A portion of each specimen was fixed in buffered formalin and sent to the Department of Pathomorphology of the University Clinical Center in Gdańsk (Medical University of Gdańsk) for further research (histological examination with immunohistochemical staining of the material). It is a generally accepted procedure during the diagnosis of neoplasms provided by the diagnostic protocol for patients with suspected neoplasms in the head and neck area.

### 4.2. Saliva Samples Collection

The collection of biological material (saliva) in the control group and the research group was performed by extracting the saliva accumulated in the oral cavity into a sterile tube by spitting the saliva directly into the tube. The above-described method of collecting biological material is a non-invasive method, the execution of which is painless, quick, and does not cause discomfort or other damage to the patient’s health. The collected biological material does not require fixation. The samples were frozen at −20 °C immediately after collection and stored until peptide extraction.

### 4.3. Isolation of Peptide Fractions from Salivary Gland Tissue

**Figure 4:**
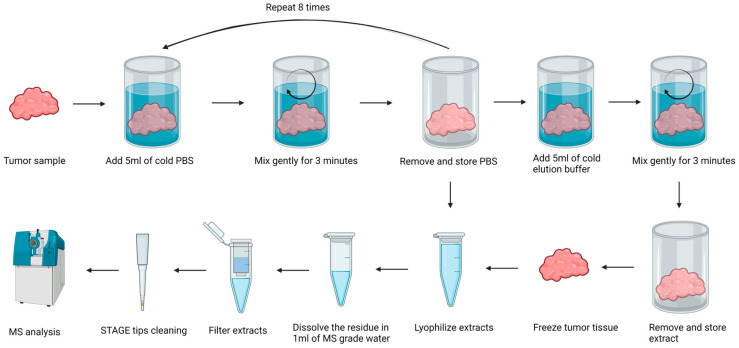
Workflow of peptide isolation from salivary gland tissue.

The general workflow of tissue sample handling is shown in Figure 4. Every tissue sample was processed with two steps of peptide elution. At first, samples were sliced into 5–10 small pieces to increase the elution surface. Then, the tissue was transferred to glass bottles and washed with 5 mL of ice-cold PBS. After 3 min of gently rinsing, PBS was collected and stored at −20 °C as PBS Fraction 1. The whole procedure was repeated up to 8 times until all traces of blood were removed. The next step after PBS washing was peptide elution. Samples were eluted with 0.1% TFA in MS-grade water (called TFA). Then, 5 mL of the appropriate, ice-cold elution solutions were added to the tissue and incubated with mixing for 3 min on ice. After that, the elution solution was removed and centrifuged for 5 min at 500× *g*, 4 °C. The supernatant was collected and stored at −20 °C as TFA extract. A total of 36 extracts were obtained from clinical samples taken from 14 different patients (Table 1). Frozen elution samples were lyophilized using CentriVap Cold Trap (Labconco, Kansas City, MO, USA). After that, lyophilisates were dissolved in 1 mL of MS-grade water and filtrated on Amicon filters with 10 kDa NMWCO (Merck Millipore, Burlington, MA, USA). The flow-through fractions containing peptides were collected. The concentration of peptides was measured with a Multiskan SkyHigh Microplate Spectrophotometer (Thermo Scientific, Waltham, MA, USA) and 10 µg of every sample was desalted in STAGE (Stop And Go Extraction) TIPS procedure [55] using Empore C18 extraction disks (3 M) with elution by 60% acetonitrile/1% acetic acid solution. Eluates were concentrated in a SpeedVac to 25 µL volume and stored at −20 °C for further LC-MS analysis.

### 4.4. Peptide Isolation from Saliva

Peptides were isolated from saliva by two methods. In the first (“Direct method”) method, 500 µL of saliva was filtered on Amicon filters with 10 kDa NMWCO. The concentration of peptides in the flow-through fraction was measured and 10 µg of every sample was desalted in STAGE TIPS procedure using Empore C18 extraction disks (3 M) with elution by 60% acetonitrile/1% acetic acid solution. Eluates were concentrated in a SpeedVac to 25 µL volume and stored at −20 °C for further LC-MS analysis.

The second method was peptide isolation by solid phase extraction (SPE). First, 100 µL of saliva was diluted in 900 µL of TFA (0.1 or 0.5%) and incubated on ice for 5 min. After incubation, all samples were centrifuged for 5 min at 4 °C and 5000× *g*. Samples were transferred to new tubes, diluted with 0.2% formic acid in a 1:1 ratio, mixed intensively for 20 s, and filtered on Amicon filters with 10 kDa NMWCO. The flow-through fraction proceeded on SupelcleanTM SPE tubes (LC-18 or HLB) (Supelco, Bellefonte, PA, USA) as previously described [56] and eluted with 500 µL 50% ACN/0.2% FA. The solvent was evaporated in a SpeedVac to complete dryness, resuspended in 30 µL of 0.1% FA, and stored at −20 °C for further LC-MS analysis.

### 4.5. LC-MS/MS

Liquid chromatography–tandem mass spectrometry measurements were performed using the Triple TOF 5600+ mass spectrometer with DuoSpray Ion Source (SCIEX, Framingham, MA, USA) coupled with Ekspert MicroLC 200 Plus (Eksigent, Dublin, CA, USA) similar to the previously described [57]. The column used for chromatographic separation was ChromXP C18CL (3 μm, 120 Å, 150 × 0.3 mm; Eksigent, Dublin, CA, USA) and the sample injection volume was 5 µL. Mobile phase A used in chromatography was 0.1% formic acid in water, and mobile phase B was 99.9% acetonitrile/0.1% formic acid. The gradient was 11% B for 2 min, then 48 min gradient to 43.5% B, 2 min gradient to 98% B, 98% B for 2 min, drop to 11% B, and 11% B for 4 min in total flowrate 5 µL/min. The column temperature was stabilized at 35 °C. The mass spectrometer operated in data-dependent acquisition (DDA) mode. MS1 scan was acquired in the mass range of 400–1200 Da with an accumulation time of 100 ms and followed by MS2 scan in the range of 100–1800 Da with an accumulation time of 50 ms. The raw data from the mass spectrometer were converted to a .mzML file with MSConvert softwarev. 3.0.22251-0c5a298 (ProteoWizard ProteoWizard, Palo Alto, CA, USA then processed in PEAKS Studio software Xpro ver. 10 (Bioinformatics Solutions Inc. Waterloo, ON, Canada) [58] and searched against the Homo sapiens OpenProt 1.6 database (May 2021) [48]. The mass spectrometry proteomics data have been deposited to the ProteomeXchange Consortium via the PRIDE [59] partner repository with the data set identifier PXD038985.

### 4.6. Exploratory Data Analysis

Exploratory Data Analysis (EDA) of peptide sequences was performed by using custom scripts written in Python programming language (ver. 3.8.8) [60]. Loading, filtering, and cleaning of data was achieved by using NumPy (ver. 1.19.5) [61] and pandas (ver. 1.1.1) [62] libraries. To compare sequences (of peptides) to each other, built-in functions/methods and data structures from the standard library were applied. Jupyter-notebook (ver. 6.1.3) was used as an environment for computations and result validation.

### 4.7. Ethics Committee Approval and Personal Data Handling

The Regional Bioethics Committee approved the research protocol (NKBBN/308/2021). Written informed consent was obtained from each participant. Each participant could opt out of the study at any stage without any consequences. The obtained biological material was placed in sterile containers with a tight lid and marked with a number corresponding to the order of obtaining during the study, which allowed for segregation of the material and conduct of analysis and correlation with clinical data. Personal data have been classified and not placed on the containers used.

## Figures and Tables

**Table 1:** Number of samples analyzed in this work with information about sample handling (fresh or frozen) or peptide extraction method used (PBS or TFA). For details see Materials and Methods section.

Salivary Gland Tissue Samples					
	Fresh		Frozen		Total
	PBS	TFA	PBS	TFA	
Tumor	5	4	5	4	18
Control	5	4	5	4	18
Total	10	8	10	8	36
**Saliva Samples**					
	Patient		Control		Total
	11		8		19

**Table 2:** Number of peptides identified in all analyses with the distinction of unique and stand-alone peptides (see text for details).

Data Sets	Peptides	Unique Peptides	Stand-Alone Peptides
SG tumor	8233	3916	1733
SG control	3515	1586	589
SG fresh	6752	3205	1383
SG frozen	6913	3212	1496
SG PBS	7771	3649	1667
SG TFA	3540	1770	702
SG All	10,380	4869	2077
Saliva patient	3201	1166	325
Saliva control	2461	836	258
Saliva all	4189	1490	420

SG—salivary gland; SG tumor—peptides identified in extracts from SG tumor tissue; SG control—peptides identified in extracts from SG healthy tissue; SG fresh—peptides identified in extracts from fresh samples of SG tissue (tumor and control); SG frozen—peptides identified in extracts from frozen samples of SG tissue (tumor and control); SG PBS—peptides identified in extracts from samples of SG tissue (tumor and control, fresh and frozen) using PBS; SG TFA—peptides identified in extracts from samples of SG tissue (tumor and control, fresh and frozen) using water with TFA; SG all—all peptides isolated from all SG samples. Saliva patient—peptides identified in patients’ saliva; Saliva control—peptides identified in control group saliva; Saliva all—peptides identified in all saliva samples (patient and control).

**Table 3:** Comparison of the numbers of peptides unique to a given group of samples.

Data Sets	Overlapping Sequences	Common Peptides
Saliva patient—SG tumor	377	86
Saliva patient only—SG tumor only	109	16
Saliva patient—SG control	960	182
Saliva patient only—SG control only	362	42
Saliva control—SG tumor	425	121
Saliva control only—SG tumor only	154	39
Saliva control—SG control	950	209
Saliva control only—SG control only	351	58

See the description of data sets in Table 2. Word “only” added to data sets means that the given data set contains peptides unique for a given group. For example, the “Saliva patient only” data set was built by removing from the data set “Saliva patient” (see Table 2) all peptides present in the data set “Saliva control” (see Table 2).

**Table 4:** A list of proteins that are the source of peptides observed only in patients’ saliva samples and extracts from tumor tissue samples (see Table 3).

Protein Accession	Protein Name	Gene Names	No. Peptides
P63261, P60709	Actin, cytoplasmic	ACTB, ACTG	9
P01009	Alpha-1-antitrypsin	SERPINA1	1
P04083	Annexin A1	ANXA1	3
V9GYC1	Apolipoprotein A-II	APOA2	1
P04280, P02812, Q04118, P10163	Basic salivary proline-rich proteins	PRB1/4	32
Q96DR5	BPI fold-containing family A member 2 (SPLUNC2, Parotid secretory protein)	BPIFA2	1
P04080	Cystatin-B	CSTB	1
P02671	Fibrinogen alpha chain	FGA	2
P02675	Fibrinogen beta chain	FGB	2
P09211	Glutathione S-transferase P	GSTP1	3
P04406	Glyceraldehyde-3-phosphate dehydrogenase (GAPDH)	GAPDH	4
P04792	Heat shock protein beta-1	HSPB1	2
P69905, P68871	Hemoglobin	HBA1/HBA2, HBB	5
P16402, P10412, P16401, P20671	Histones	H1-3, H1-4, H1-5, H2AC7	9
P01857	Immunoglobulin heavy constant gamma 1	IGHG1	1
P04264, P13647	Keratin	KRT1, KRT5	4
Q09666	Neuroblast differentiation-associated protein AHNAK (Desmoyokin)	AHNAK	2 *
P05109	Protein S100-A8 (Calgranulin-A)	S100A8	2
P06702	Protein S100-A9 (Calgranulin-B)	S100A9	2
Q08188	Protein-glutamine gamma-glutamyltransferase E	TGM3	1
P02810	Salivary acidic proline-rich phosphoprotein 1/2	PRH1; PRH2	17
P62328	Thymosin beta-4	TMSB4X	2 (1 **)
P60174	Triosephosphate isomerase	TPI1	2
P08670	Vimentin	VIM	1

In red—proteins previously linked with salivary gland tumors; in yellow—proteins previously linked with other cancer types; *—peptides of isoform origin (OpenProt); **—peptides of pseudogene origin (OpenProt).

## Data Availability

The mass spectrometry proteomics data have been deposited to the ProteomeXchange Consortium via the PRIDE partner repository with the data set identifier PXD038985.

## Supplementary Materials

The following supporting information can be downloaded at: https://www.mdpi.com/article/10.3390/ijms25168799/s1.
